# A Combination of Caffeine, TeaCrine® (Theacrine), and Dynamine® (Methylliberine) Increases Cognitive Performance and Reaction Time Without Interfering With Mood in Adult Male Egamers

**DOI:** 10.7759/cureus.20534

**Published:** 2021-12-20

**Authors:** Jaime L Tartar, Jonathan B Banks, Mykola Marang, Frankie Pizzo, Jose Antonio

**Affiliations:** 1 Psychology and Neuroscience, Nova Southeastern University, Davie, USA; 2 College of Health Care Sciences, Nova Southeastern University, Davie, USA

**Keywords:** teacrine, egamer, dynamine, cognition, caffeine, attention

## Abstract

Background

Involvement in video game activities and competitive video gaming (esports) is a rapidly growing field. Moreover, there is a marked interest in identifying nutritional supplements to safely improve egamer performance.

Methodology

We conducted a repeated-measure, randomized crossover study to compare the effects of caffeine (125 mg), caffeine (125 mg) + Dynamine® (75 mg) + TeaCrine® (50 mg) (CDT), and matched placebo across three testing sessions (one week apart) among 50 young male egamers. We tested the effect of each product on multiple measures of cognition, self-reported mood (anxiety, alertness, and headache), and biomarkers of arousal (cortisol and salivary alpha-amylase). We also measured electroencephalogram power during the cognitive tasks. Finally, we tested whether individual differences in xenobiotic metabolism would affect the study outcome measures by genotyping each participant for cytochrome P450 1A2*1F (CYP1A2*1F) allele status.

Results

Compared to pre-dose, CDT improved performance on the Flanker Test of Inhibitory Control and improved reaction time on the Psychomotor Vigilance Task post-dose. Compared to the placebo, caffeine increased self-reported anxiety whereas the CDT combination increased self-reported alertness. Compared to the CDT combination, caffeine increased self-reported headaches. Physiological measures suggested that increases in delta EEG power and cortisol production are associated with the effects observed in the CDT condition to optimize certain aspects of egamer performance. CYP1A2*1F allele status did not moderate outcome variables between conditions in this study.

Conclusions

CDT is a safe and effective product for improving cognitive performance among egamers without increasing self-reported anxiety or headaches. EEG changes demonstrate that CDT increased attention to internal processing (i.e., increased cortical delta power) and potentially increased cognitive control (i.e., increased cortical theta frequency), while the increases in cortisol suggest increased energy mobilization. Future work should aim to clarify the physiological underpinnings of CDT-induced changes in performance and examine the effects of CDT under naturalistic egamer conditions.

## Introduction

Involvement in video game activities and competitive video gaming (esports) is a rapidly growing field. Esport activities involve participation, competition, and viewing parallel traditional sports [1]. While seemingly passive activity, many video games require a high level of cognitive control, cognitive flexibility, and heightened vigilance. Related to these demands, experienced video game players (egamers) outperform non-video game players on multiple measures of cognitive functioning [2-5]. In fact, cognitive improvements observed among expert video game players reflect the frequent game-specific activation of cognitive processes [6]. Due to the high cognitive load associated with amateur gaming and esports, it is common for egamers to use ergogenic aids to increase their performance.

Caffeine is a widely used cognitive enhancer and is regularly used by egamers to increase their alertness, vigilance, attention, and reaction time. Previous work has shown that caffeine levels of 3 mg/kg of body weight can improve reaction times in first-person shooter games [7]. In general, caffeine in the range of 32 to 300 mg improves attention, vigilance, and reaction time [8-11]. The cognitive performance-enhancing effects of caffeine are attributed to its antagonistic role of adenosine A1 and A2A receptors in dopamine-rich brain regions. This results in increased dopamine, norepinephrine, and glutamate release which behaviorally leads to increased wakefulness and enhanced motor activity [12-14]. However, high doses of caffeine are associated with several undesirable side-effects such as increased tension and jitteriness which can impair video game performance [15,16]. In addition, caffeine, especially close to bedtime, decreases total sleep time and specifically reduces deep sleep which can impair long-term gaming performance [17-20].

Due to the unwanted side-effects of high doses of caffeine, there is an interest in identifying nutritional supplements to improve egamer performance that can complement or replace caffeine. A previous study among adult egamers showed that an inositol-enhanced arginine silicate oral supplement (nooLVL®, Nutrition 21 Purchase, NY, USA) acutely decreased error rates and completion time on the Trail Making Test. Moreover, it improved the performance of egamers on the Stroop task and improved mood acutely and after seven days of continuous use [21]. Theacrine (1,3,7,9-tetramethyluric acid) is another possible non-caffeine supplement to improve egamer performance. Theacrine is a pure alkaloid isolated from the Kucha tea leaf and other plant species, and, like caffeine, is an adenosine receptor antagonist and activates dopamine D1 and D2 receptors [22,23]. TeaCrine® (pure theacrine; Compound Solutions, Inc., Carlsbad, CA, USA) also serves as a prospective supplement to improved egamer performance given its ability to improve the subjective ratings of energy, focus, and concentration [24,25]. However, a study involving soccer players showed that TeaCrine combined with caffeine only conferred modest cognitive benefits [26]. Similar to TeaCrine®, methylliberine (2-methoxy-1,7,9 tetramethyluric acid) is also a purine alkaloid. Methylliberine is a metabolite of caffeine that is also isolated from the Kucha tea leaf and acts at adenosine receptors [27]. As a newer supplement, research on methylliberine (Dynamine®, Compound Solutions, Inc., Carlsbad, CA, USA) in isolation is limited. A safety study among humans showed that four weeks of Dynamine® supplementation at a low (100 mg) or a high dose (150 mg) did not affect biomarkers of health [28]. Although research on the effect of Dynamine® alone on cognition is limited, a previous study showed that a combination of methylliberine and caffeine works synergistically to improve self-reported mood [29].

Furthermore, a recent study among egamers also showed a potential synergistic effect of caffeine, TeaCrine®, and Dynamine® [30]. In this double-blind, placebo-controlled, crossover study, participants ingested a capsule containing either placebo, caffeine (125 mg) alone, or a combination of caffeine (125 mg) + Dynamine® (75 mg ) + TeaCrine® (50 mg) (CDT). The participants completed a first-person shooter game developed by Aim Lab (State Space Labs, Inc., New York, USA) and a visual analog scale (VAS) for the self-reported mood before and at three time points after ingesting the study product. The results showed that compared to placebo, the CDT combination improved various aspects of player performance, including shooting accuracy, visuospatial working memory, speed, and perceived (self-assessed) performance. Although caffeine also improved various aspects of player performance, it also increased perceived “jitteriness” while the CDT combination did not [30].

The current study aimed to follow up on these results by testing egamers on traditional cognitive tasks under three conditions: placebo, caffeine alone, and caffeine combined with the non-caffeine adjunctive supplements Dynamine® and TeaCrine® (i.e., CDT). We hypothesized that CDT would improve neurobehavioral performance without increasing jitteriness. As a secondary aim, we tested the effects of the three treatment conditions on the biochemical measures of stress and physiological arousal, including heart rate, blood pressure, salivary cortisol and alpha-amylase (sAA), and electroencephalogram (EEG) frequency bands. Finally, to take into consideration individual differences in xenobiotic metabolism, we genotyped participants on a single nucleotide polymorphism (SNP) on the *cytochrome P450 1A2* (*CYP1A2*) gene.

## Materials and methods

Study participants

We tested 50 male amateur egamers who self-reported playing a first-person video game for at least 10 hours a week (mean age = 20.52 years, SD = 2.03; average height = 177.63 cm, SD = 9.11; and average weight = 81.67 kg, SD = 17.18). This was a randomized, double-blinded, crossover (counterbalanced for order effect) study. Participants were tested in three testing sessions that were one week apart (see Table 1 for study design). The study conditions included caffeine (125 mg), caffeine (125 mg) + TeaCrine® (50 mg) + Dynamine® (75 mg) (CDT), and matched placebo. In this study, we included males who were 18 to 40 years old; maintained a stable lifestyle with no change in exercise, diet, or gaming during the duration of the study; regular consumers of caffeine either through caffeinated beverages or foods at a level of less than 400 mg/day; and non-smokers. Exclusion criteria included a known sensitivity or allergy to any of the study products or their ingredients, having any medical condition or disease that might impact the administration of energy supplements, having a history of drug or alcohol abuse in the 12 months prior to the study, having a history of psychiatric illness requiring hospitalization in the six months prior to screening, having a history of diabetes (type I or II) or uncontrolled hypertension (either systolic blood pressure [BP] >160 or diastolic BP >90), and participation in or use of a research product within 30 days prior to the current study. All participants underwent an informed consent process in accordance with the Declaration of Helsinki and an approved IRB protocol submitted to the institutional review board of Nova Southeastern University (approval number: 2020-116-NSU).

**Table 1:** Overview of the study protocol. BIA: bioelectrical impedance analysis; sAA: salivary alpha-amylase; MAPSS: Mood, Alertness and Physical Sensations Scales; RHR: resting heart rate; BP: blood pressure

Visit one (day 1)	Visit two (day 8)	Visit three (day 15)
Informed consent	-	-
Order randomization	-	-
Demographics	-	-
In-body (BIA) physical characteristics of participants	-	-
Baseline saliva collection (sAA and cortisol) including sample collection for genotyping	Baseline saliva collection (sAA and cortisol)	Baseline saliva collection (sAA and cortisol)
MAPSS	MAPSS	MAPSS
Cognition testing	Cognition testing	Cognition testing
Post-cognition baseline saliva collection (sAA and cortisol)	Post-cognition baseline saliva collection (sAA and cortisol)	Post-cognition baseline saliva collection (sAA and cortisol)
On-site dosing	On-site dosing	On-site dosing
60-minute wait	60-minute wait	60-minute wait
RHR/BP	RHR/BP	RHR/BP
Post-dose saliva collection (sAA and cortisol)	Post-dose saliva collection (sAA and cortisol)	Post-dose saliva collection (sAA and cortisol)
MAPSS	MAPSS	MAPSS
Cognition testing	Cognition testing	Cognition testing
Post-dose, post-caffeine saliva collection (sAA and cortisol)	Post-dose, post-caffeine saliva collection (sAA and cortisol)	Post-dose, post-caffeine saliva collection (sAA and cortisol)

Neurobehavioral performance and mood measures

Psychomotor Vigilance Test

The Psychomotor Vigilance Test (PVT) task used in this study was developed by Joggle Research and is administered on an iPad app [31]. This test measures an individual’s sustained attention through behavioral alertness. It is based on simple reaction time to stimuli that occur at random intervals and therefore measures vigilant attention [32]. False starts are also measured. The PVT is among the most widely used measures of behavioral alertness.

Flanker Inhibitory Control and Attention Test

The flanker task used in this study is part of the National Institutes of Health (NIH) toolbox cognition measures administered on an iPad app [33]. The flanker task measures attention and inhibitory control. The test requires the participant to focus on a given stimulus while inhibiting attention to stimuli (arrows) flanking it. Sometimes the middle stimulus is pointing in the same direction as the “flankers” (congruent) and sometimes in the opposite direction (incongruent). Scoring is reported as normalized t scores and is based on a combination of accuracy and reaction time.

Pattern Comparison Processing Speed Test

The pattern comparison test used in this study is part of the NIH toolbox cognition measures administered on an iPad app [33]. The pattern comparison test assesses an individual’s ability to quickly process information. It measures the amount of information that can be processed within a certain unit of time. Items are simple, and the test was developed to measure processing speed. Scoring is reported as normalized t scores and is based on a combination of accuracy and reaction time.

Dimensional Change Card Sort Test

The Dimensional Change Card Sort Test (DSST) used in this study is part of the NIH toolbox cognition measures administered on an iPad app [33]. The DSST is a test of executive function and measures the capacity to plan, organize, and monitor executive behaviors that are strategically directed in a goal-oriented manner. Scoring is reported as normalized t scores and is based on a combination of accuracy and reaction time.

Mood, Alertness, and Physical Sensations Scales

The Mood, Alertness, and Physical Sensations Scales (MAPSS) was developed specifically for use in caffeine studies [34] and includes 19 items (single or groups of words/descriptors) describing moods and physical sensations, which are rated on a nine-point scale anchored at the left-hand end with “not at all” and the right-hand end with “extremely.” MAPSS includes three subscales based on Alertness, Anxiety, and Headache [35].

Physiological measures of stress and arousal

Neurophysiological Arousal

Measures of neurophysiological arousal during cognitive task performance were also assessed. We used a non-invasive, wearable, single-channel EEG (Enchanted Wave, LLC, Florida, USA). The Enchanted Wave EEG device is an ambulatory, wireless sleep staging tool that includes a headband containing two dry electrodes which record signals from the forehead at the Fp1 region based on the 10-20 system of electrode placement. Alongside these sensors are two metallic fabric electrodes by the mastoid bone which require skin contact. At the end of each recording, the device’s automated software carries out a fast Fourier transformation algorithm. Data are sampled every four seconds, and we averaged EEG band signals and band frequencies across each recording session.

Biomarkers of Arousal

sAA is an index of fast-responding sympathetic nervous system activity and correlates with norepinephrine, while cortisol is a marker of the slower-acting hypothalamic-pituitary-adrenal axis activity [36]. Each measure is unique, but complementary, in the ability to assess stress and nervous system activity. Saliva samples were obtained from each participant via passive drool. The samples were collected in 1.5 mL polypropylene microcentrifuge tubes and kept on ice until the session was complete. Following the session, the samples were stored in a freezer at -20°C until assayed. Saliva samples were run in duplicates. Cortisol was quantified via a human enzyme immunoassay kit, and sAA was quantified via a kinetic reaction kit according to the manufacturer’s instructions (Salimetrics, Carlsbad, CA, USA) [37]. The functional sensitivity of the salivary cortisol enzyme-linked immunosorbent assay is 0.018 μg/dL, and the sAA kinetic reaction is 2.0 U/mL.

Heart Rate and Blood Pressure

Heart rate and BP were assessed via a standard electronic blood pressure device (Walgreens deluxe arm blood pressure monitor, model number WGNBPA-230).

Genotyping

CYP1A2 Gene Single Nucleotide Polymorphisms

Saliva samples were collected at the beginning of the study in 1.5 mL polypropylene microcentrifuge tubes and kept on ice until the session was complete. Following the session, the samples were stored in a freezer at -20°C until assayed for genotyping. Genomic DNA extraction was performed using the QIAamp DNA Investigator kit in accordance with the manufacturer’s instructions (QIAGEN, Valencia, CA, USA). After isolation, amplification was conducted by real-time polymerase chain reaction (PCR) using TaqMan SNP genotyping assays using fluorogenic probes (Applied Biosystems, CA, USA) for the CYP1A2*1F (rs762551) SNP. The Applied Biosystems software automatically genotyped participants based on end-point fluorescence intensity. All samples were run in duplicates, and in the event of genotype call discrepancy, the samples were re-run.

Statistical analyses

To test the hypothesis that CDT would improve neurobehavioral performance without increasing jitteriness, relative to both the caffeine and control conditions, we conducted a series of hierarchical linear model (HLM) analyses. These analyses allowed for an examination of performance on the neurobehavioral tasks and mood ratings while controlling for the repeated-measures nature of the data. Specifically, the impact of both time (within-session changes pre- to post-administration of intervention) and condition (between-session control, caffeine, or CDT) and their interaction were modeled for each measure. In addition, HLM analyses were used to test for the effects of the three treatment conditions on heart rate, BP, cortisol, sAA, and EEG frequency bands. Cortisol and sAA changes across the session were restricted to the saliva assessments prior to the completion of the cognitive tasks (points 1 and 3). Finally, prior HLM analyses were repeated with the SNP entered as interaction terms for the effects of condition and change in performance across the session. The CYP1A2*1F SNP was evaluated independently in each model. The CYP1A2*1F SNP was collapsed to one group with CC and AC included and a second group with AA (fast xenobiotic metabolism) due to the AA SNP predicting fast xenobiotic metabolism.

## Results

Effects on cognitive performance and mood

**Figure 1:**
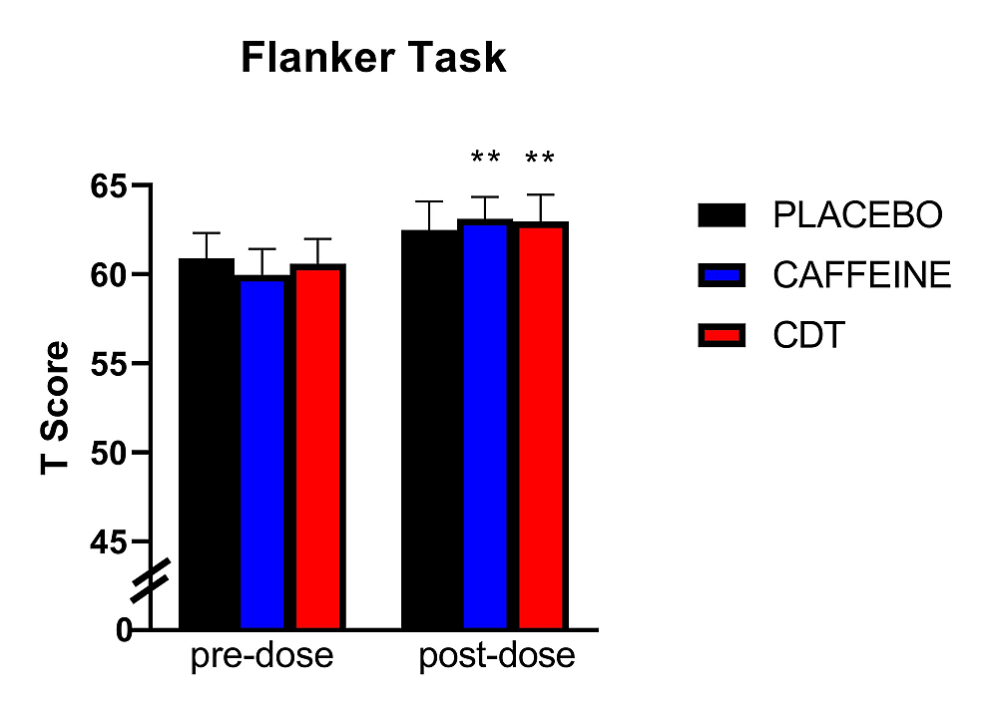
Flanker inhibitory control and attention task. Double asterisks (**) indicate a significant difference from pre-dose (p < 0.01). Error bars indicate standard error.

To test the first hypothesis that CDT would improve neurobehavioral performance we conducted a series of HLM analyses on all cognitive tasks. In the first model examining the effect of condition (control, caffeine, and CDT) and time (pre and post-dose) on flanker performance, a significant effect of time was observed [F (1, 245) = 19.50; p < 0.001)] such that scores on the flanker task increased from pre (M = 60.5, SD = 10.05) to post-dose (M = 62.9, SD = 8.31). As shown in Table 2, this increase in flanker performance was statistically significant for the caffeine group [t (49) = 3.66; p = 0.002] and the CDT group [t (49) = 3.31; p = 0.004] but not the control group [t (49) = 1.90; p = .064] (Figure 1).

**Table 2:** Means and SDs for study variables. SD: standard deviation; PVT: Psychomotor Vigilance Test; MAPSS: Mood, Alertness and Physical Sensations Scales; sAA: salivary alpha-amylase; EEG: electroencephalogram

	Control condition		Caffeine condition			CDT condition
	Pre-dose M (SD)	Post-dose M (SD)	Pre-dose M (SD)	Post-dose M (SD)	Pre-dose M (SD)	Post-dose M (SD)
Flanker	60.90 (10.01)	62.48 (8.21)	59.96 (10.41)	63.12 (8.71)	60.60 (9.89)	62.96 (8.14)
Pattern comparison	67.10 (8.77)	70.48 (4.98)	65.94 (9.50)	69.90 (5.80)	65.90 (8.89)	69.80 (5.65)
Dimensional Change Card Sort	60.20 (10.72)	61.46 (10.31)	59.42 (10.39)	60.86 (8.67)	60.54 (10.54)	61.92 (9.11)
PVT errors	1.82 (2.27)	1.96 (1.99)	1.34 (1.64)	1.74 (1.85)	1.58 (1.95)	1.50 (1.85)
PVT reaction time	272.20 (56.69)	277.28 (65.34)	268.78 (249.55)	267.91 (59.16)	283.44 (89.98)	267.45 (63.22)
MAPSS Alertness	6.59 (1.15)	6.82 (1.19)	6.79 (1.13)	7.04 (1.18)	6.98 (1.02)	7.04 (1.13)
MAPSS Anxiety	1.92 (0.80)	1.78 (0.72)	2.23 (1.38)	2.34 (1.74)	1.84 (0.99)	1.94 (1.22)
MAPSS Headache	1.45 (0.65)	1.47 (0.58)	1.75 (1.35)	1.72 (1.45)	1.37 (0.76)	1.35 (0.61)
sAA	90.25 (78.68)	98.95 (81.43)	93.94 (78.96)	111.02 (88.33)	88.44 (68.96)	94.15 (78.27)
Cortisol	0.24 (0.20)	0.14 (0.11)	0.26 (0.20)	0.15 (0.12)	0.27 (0.24)	0.26 (0.34)
EEG alpha	6.90 (6.76)	6.12 (3.61)	7.11 (5.00)	3.26 (6.45)	5.71 (2.77)	6.82 (5.84)
EEG beta	36.93 (39.18)	31.56 (17.29)	36.73 (25.88)	34.27 (45.01)	30.45 (14.19)	36.29 (40.46)
EEG delta	29.73 (21.03)	28.27 (18.23)	31.53 (20.12)	25.93 (14.91)	25.81 (13.71)	29.58 (14.94)
EEG theta	18.55 (14.25)	17.12 (9.72)	19.38 (12.49)	16.83 (12.69)	15.83 (7.32)	18.45 (10.96)

**Figure 2:**
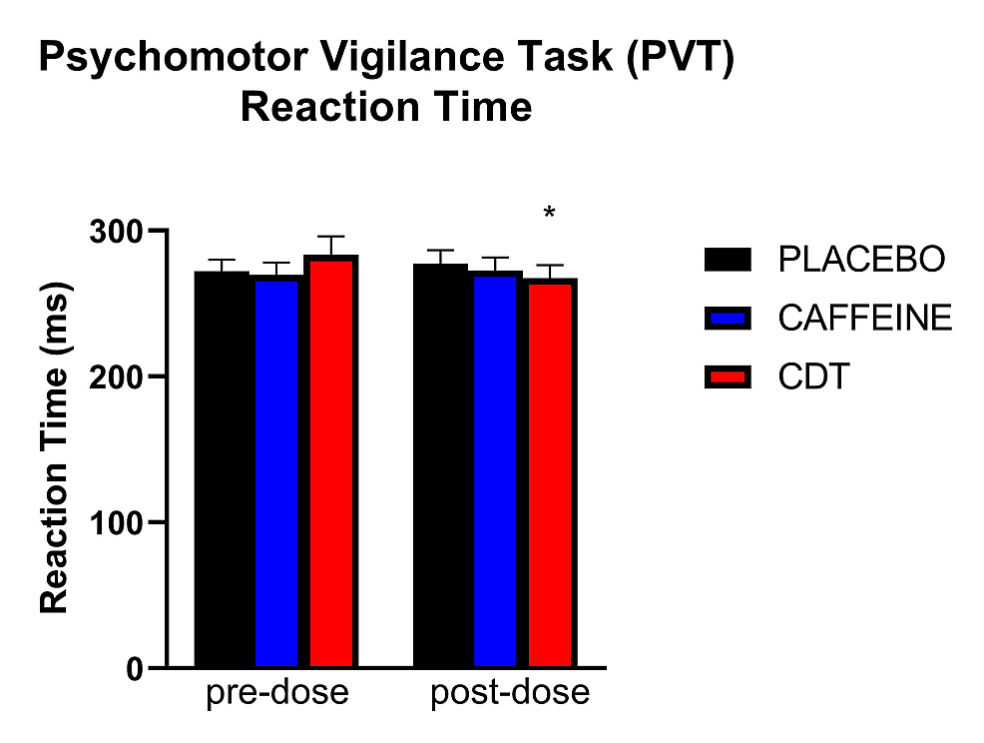
Psychomotor Vigilance Task reaction time. Asterisk (*) indicates a significant difference from pre-dose (p < 0.05). Error bars indicate standard error.

A significant effect of time, but not condition or time by condition interaction, was observed for the processing speed task [F (1, 243.85) = 39.92; p < 0.001] such that processing speed increased from pre (M = 66.3, SD = 9.02) to post-dose (M = 70.0, SD = 5.46). A trend for time by condition interaction was observed on the PVT reaction time [F (1, 244) = 2.93; p = 0.055]. Significant decreases (improvement) in reaction time were observed in the CDT condition from pre to post-dose [t (244) = 2.46; p = 0.044] (Figure 2), but not for the caffeine or control condition (p > 0.05), as shown in Table 2. No significant effect of time, condition, or time by condition interactions were observed for DSST performance or PVT performance, as measured by errors.

HLM analyses examining mood revealed a significant effect of condition but no effect of time or time by condition interaction for Alertness [F (2, 245) = 3.09; p = 0.047). Post-hoc analyses revealed a significant difference between the control condition (M = 6.70, SD = 1.17) and the CDT condition (M = 7.00, SD = 1.07) (t = 2.68; p = 0.026). Similarly, a main effect of condition but no effect of time or time by condition interaction was found for Anxiety [F (2, 245) = 6.10; p = 0.003]. Post-hoc analyses revealed a significant difference between the control condition (M = 1.85, SD = 0.76) and the caffeine condition (M = 2.29, SD = 1.57) (t = 2.83; p = 0.017). A main effect of condition but no effect of time or time by condition interaction was observed for headaches [F (2, 245) = 5.87; p = 0.003]. Post-hoc analyses revealed a significant difference between the caffeine condition (M = 1.73, SD = 1.39) and the CDT condition (M = 1.36, SD = 0.69) (t = 2.57; p = 0.012).

Effect on biomarkers and electroencephalogram

**Figure 3:**
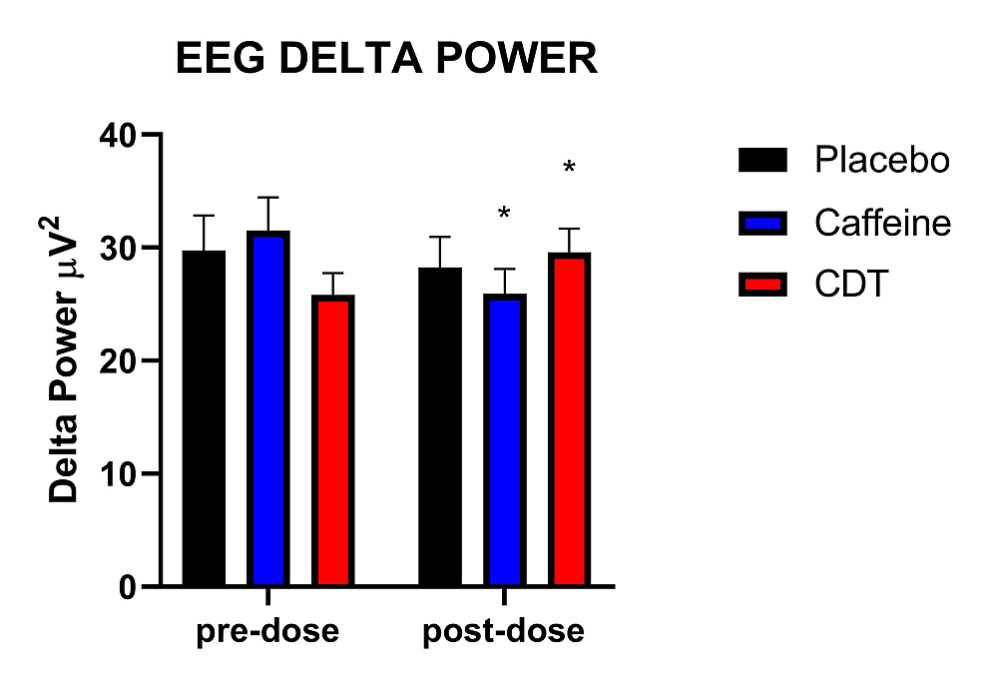
EEG delta power. Asterisks (*) indicate a significant difference from pre-dose (p < 0.05). Error bars indicate standard error. EEG: electroencephalogram

Next, we examined the impact of the condition on heart rate, BP, cortisol, sAA, and EEG frequency bands. A significant time by condition interaction was found for the delta power [F (2, 231.61) = 3.24; p = 0.041]. Post-hoc analyses revealed a significant decrease in delta power in the caffeine group from pre to post-dose [t (46) = 2.17; p = 0.035), but a significant increase in the delta band in the CDT group from pre to post-dose [t (49) = 2.01; p = 0.050) (Table 2, Figure 3). No change was observed in the control condition (p > 0.05). We found a trend for interaction between time by condition for the theta band [F (2, 231.75) = 2.20; p = 0.113]. Post-hoc analyses revealed a significant increase in theta bands in the CDT group from pre to post-dose [t (49) = 2.10; p = 0.041], but no change in the control or caffeine groups (p > 0.05). No significant time, condition, or time by condition interactions were observed for either alpha or beta bands.

The HLM analysis examining the impact of condition and time on cortisol revealed a significant effect of time [F(1, 238.05) = 15.02; p < 0.001], a significant effect of condition [F (2, 238.42) = 5.51; p = 0.005], and a trend for an interaction between time and condition [F (2, 238.05) = 2.73; p = 0.067]. When collapsed across time (pre-post), cortisol levels were lower in the control condition (M = 0.19, SD = 0.17) than the CDT condition (M = 0.27, SD = 0.29) (t = 2.48; p = 0.041). Further, cortisol levels decreased from pre (M = 0.26, SD = 0.21) to post measurement (M = 0.18, SD = 0.23) (t = 2.93; p = 0.004). However, the change over sessions was only significant for the control condition (t = 2.98; p = 0.010) and the caffeine condition (t = 3.37; p = 0.003). Cortisol levels did not change across the session in the CDT condition (t = 0.349; p = 1.00) (Table 2). No effect of time, condition, or time by condition interaction was observed for sAA.

Moderating effects of genotype

When SNP CYP1A2*1F (rs762551) was added as an interaction term to the model predicting speed of processing, a significant interaction between the SNP CYP1A2*1F and condition was observed [F (2, 238.86) = 3.16; p = 0.044]. Post-hoc analyses did not reveal significant differences. There was no effect of genotype between conditions. Based on the question of interest in the study (i.e., pre to post changes within each session by condition) and the SNP by condition effects observed on the speed of processing task, we examined changes across the session for each condition. A significant increase in the speed of processing was observed in the control condition among the C/- allele carriers (pre M = 65.2, SD = 12.30; post M = 71.2, SD = 7.17) (t = 3.83; p = 0.011), but not in the control condition among the AA (fast metabolism) allele carriers (pre M = 68.3, SD = 5.20; post M = 70.0, SD = 2.74; p > 0.05). Further, a significant increase in the speed of processing was observed in the caffeine condition among the AA (fast metabolism) allele carriers (pre M = 64.6, SD = 9.38; post M = 69.1, SD = 4.33) (t = 3.47; p = 0.039), but not among the C/- allele carriers (pre M = 67.9, SD = 9.58; post M = 71.2, SD = 7.44; p > 0.05).

On examining the impact of genotype on the DSST performance, a significant interaction between time and CYP1A2*1F was observed [F (1, 240) = 5.09; p = 0.025]. Post-hoc analyses revealed that card sort performance improved across the session (pre to post) for the AA (fast metabolism) allele carriers (pre M = 58.7, SD = 9.56; post M = 61.4, SD = 8.35) (t = 2.89; p = 0.009), but not among the C/- allele carriers (pre M = 62.1, SD = 11.52; post M = 61.5, SD = 10.73; p > 0.05). CYP1A2*1F allele status did not alter the findings for alpha, beta, theta, delta, cortisol, or sAA.

## Discussion

This repeated-measures, randomized crossover study assessed the effects of caffeine, CDT, and placebo conditions across three testing sessions in a sample of young male egamers. We tested the effect of each condition on multiple measures of cognition, self-reported mood, and biomarkers of arousal (cortisol and sAA). In addition, we measured EEG power during cognitive tasks. Finally, we tested whether individual differences in xenobiotic metabolism would affect the study outcome measures by genotyping each participant for CYP1A2*1F allele status.

On the measures of cognition, we found that, relative to caffeine, both CDT and placebo improved performance on the Flanker Test of Inhibitory Control and Attention. The flanker task measures both a participant’s attention and inhibitory control. Previous studies have shown that caffeine improves flanker task performance in a dose-response manner but asymptotes at 200 mg [38]. However, other studies showed that a 3 mg/kg dose of caffeine does not significantly reduce interference on the flanker task [39,40]. Our findings suggest that Dynamine® and TeaCrine® might augment the effects of caffeine on flanker task performance. TeaCrine® and Dynamine® are thought to act through both the adenosine and dopamine systems and flanker task performance has been shown to improve with transcranial direct current stimulation of the left dorsolateral prefrontal cortex (DLPFC) [41]. It is possible that CDT augments the attention and inhibitory control on flanker performance because dopaminergic modulation of the DLPFC is critical for attention and inhibition [42]. In addition to improving flanker performance, the CDT condition resulted in a significant decrease in PVT reaction time. The PVT is among the most widely used measures of behavioral alertness. While previous studies have reported that 60 mg of caffeine significantly improves PVT performance [43-45], we did not replicate this finding here. However, we did find that the CDT condition resulted in faster reaction time, suggesting that the combination of caffeine, TeaCrine®, and Dynamine® (CDT) might work synergistically to improve vigilant attention. There were no significant effects of any treatment condition on PVT errors, processing speed, or dimensional change card sort task performance (a measure of executive function). This is in contrast to previous studies reporting that caffeine improves response times during task switching [46] as well as improves general performance on the card sort task [47]. It is possible that the differences in findings are due to examining the overall reaction time in the current study compared to improved switch times in previous studies. Additionally, the card sort task in this study was delivered on a different platform (NIH toolbox) compared to prior work, thus variations in the task may play a role in the different results.

For the self-reported mood measures, the CDT condition resulted in a significant increase in alertness relative to the control condition. There was also a significant increase in self-reported anxiety in the caffeine condition relative to the placebo condition. While caffeine is known to have anxiogenic effects [48], it is notable that caffeine when combined with TeaCrine® and Dynamine® does not result in increased anxiety compared to placebo. A previous study conducted among mice suggested that TeaCrine® can have a calming effect on the nervous system and can counteract caffeine-induced insomnia [49]. Accordingly, it is possible that a neutralizing effect of caffeine-induced anxiety occurred in this study. Finally, relative to the caffeine condition, the CDT condition resulted in a lower score for self-reported headaches. While it is unclear why CDT would reduce headaches relative to caffeine alone, it is possibly related to the modulation of dopamine that is unique to CDT. Indeed, the adenosine A2A receptor subtype is widespread in the areas of the brain that are innervated by dopamine fibers (e.g., striatum, nucleus accumbens, and olfactory tubercle) [50], and modulation of dopamine has previously been shown to mitigate headache and migraine symptoms [51].

EEG was recorded during the cognitive tasks during each condition. There was a significant decrease in delta power in the caffeine group (pre to post-dose), while there was a significant increase in delta power in the CDT group (pre to post-dose). The differential effects of caffeine versus CDT are possibly due to the activity of TeaCrine® and Dynamine® at adenosine receptors. Caffeine, TeaCrine®, and Dynamine® act at adenosine A1 receptors that are abundant in the cortex where electrical patterns are prominently recorded during EEG [52]. It is possible that because TeaCrine® and Dynamine® are allosteric modulators of adenosine receptors while caffeine is a competitive inhibitor, this differential activity at cortical A1 receptors is associated with the differential delta activity between treatments. In general, increased delta activity during a cognitive task can reflect an increase in attention to internal cognitive processes [53]. While we did not find significant treatment effects across conditions in other EEG band frequencies, there was a trend for theta power in the omnibus analysis, and a follow-up post-hoc analysis showed a significant increase (pre to post) in theta bands in the CDT group. Given that increased theta power is associated with increased cognitive control [54], future research should investigate the extent to which CDT can influence theta activity among egamers. Changes in EEG activity that reflect improved cognitive function or effort are relevant to egamers, in particular, given that this group shows higher levels of cognitive performance relative to non-gamers [2-5].

Biomarker analyses revealed that cortisol levels were significantly increased in the CDT condition relative to the control condition. In addition, cortisol levels only significantly decreased in the control and caffeine conditions pre to post-dose. It is somewhat surprising that the caffeine condition decreased cortisol levels because caffeine is known to induce the release of cortisol from the adrenal cortex [55]. Although the ability of TeaCrine® and Dynamine® to induce the release of cortisol is currently unclear, the increase in cortisol in the CDT group possibly reflects increased energy mobilization with this treatment. Future work should aim to examine the ability of these products to induce cortisol release in humans. There were no effects of treatment on sAA levels.

Furthermore, we assessed the ability of the CYP1A2*1F (rs762551) SNP to moderate study outcome variables between treatment conditions. This SNP is associated with increased enzymatic breakdown of xenobiotics. In particular, AA homozygotes have been shown to have faster caffeine metabolism, and C/- genotypes show normal metabolism of caffeine [56]. In this study, we did not find any condition by genotype relationships. In other words, there does not appear to be an effect of individual differences on product breakdown regarding any of the outcome variables in the study. There was an effect of genotype pre to post-dose on caffeine for the speed of processing. In general, there was only a 60-minute period between dosing and testing. It is possible that the CYP1A2*1F SNP could affect cognition performance, mood, or biomarker levels between conditions at later time points. In the context of the present study, metabolism differences due to CYP1A2*1F allele status do not appear to functionally matter for performance differences between CDT and caffeine.

The current study has several limitations. First, this study was limited to a male egamer population. Accordingly, we do not know if any of the study effects would replicate among women. Second, as the study participants were young and healthy, we cannot be sure what effects CDT would have on any study variables in older adults or a population that is cognitively vulnerable. Finally, the condition under which the participants were tested was a quiet, stress-free testing environment that does not replicate some gaming environments. Future work should aim to replicate study variables in a typical gaming environment (i.e., head-to-head competition).

## Conclusions

The current study found that CDT is a safe and effective product for improving cognitive performance among egamers without increasing self-reported anxiety or headaches. The EEG changes in this study demonstrate that CDT increased attention to internal processing (i.e., increased cortical delta power) and potentially increased cognitive control (i.e., increased cortical theta frequency), while the increases in cortisol suggest increased energy mobilization. Future work should aim to further clarify these relationships.
